# Targeting the receptor tyrosine kinase MerTK shows therapeutic value in gastric adenocarcinoma

**DOI:** 10.1002/cam4.6866

**Published:** 2024-03-28

**Authors:** Naita Maren Wirsik, Mingyi Chen, Liping He, Uraz Yasar, Justus Gaukel, Alexander Quaas, Henrik Nienhüser, Ella Leugner, Shuai Yuan, Nikolai Schleussner, Jin‐On Jung, Jadie Sue Plücker, Martin Schneider, Thomas Schmidt

**Affiliations:** ^1^ Department of General, Visceral and Transplantation Surgery University of Heidelberg Heidelberg Germany; ^2^ Department of General, Visceral and Transplantation Surgery University Hospital Cologne Cologne Germany; ^3^ Institute of Pathology University Hospital Cologne Cologne Germany

**Keywords:** chemotherapy, gastric adenocarcinoma, MerTK, survival, UNC2025

## Abstract

**Background:**

Despite multiple therapeutic modalities, the overall survival of patients with gastric adenocarcinoma remains poor, especially for advanced tumor stages. Although the tyrosine kinase MerTK has shown therapeutic relevance in several tumor entities, its potential effects in gastric adenocarcinoma have not yet been sufficiently characterized.

**Methods:**

MerTK expression and its influence on patient survival were evaluated by immunohistochemistry in a cohort of 140 patients with gastric adenocarcinoma. CRISPR/Cas9 knockout and siRNA knockdown of MerTK in the gastric cancer cell lines SNU1, SNU5, and MKN45 was used to analyze protein expression, growth, migration, and invasion properties in vitro and in a murine xenograft model. MerTK was pharmacologically targeted with the small molecule inhibitor UNC2025 in vitro and in vivo.

**Results:**

In patients, high MerTK expression was associated with decreased overall survival (OS) and lymph node metastasis especially in patients without neoadjuvant therapy (*p* < 0.05). Knockout and knockdown of MerTK reduced cell proliferation and migration both in vitro and in vivo. UNC2025, a small‐molecule inhibitor of MerTK, exhibited a significant therapeutic response in vitro and in vivo. Additionally, MerTK expression attenuated the response to neoadjuvant treatment, and its inhibition sensitized tumor cells to 5‐Fluorouracil (5‐FU)‐based chemotherapy in vitro.

**Conclusions:**

Our findings demonstrate the potential value of MerTK as a prognostic biomarker for gastric adenocarcinoma. Targeting MerTK may become a new treatment option, especially for patients with advanced tumors, and may overcome resistance to established chemotherapies.

## Graphical abstract

Evaluating the role of tyrosine kinase Mer in gastric adenocarcinoma and the possible value of a specific inhibition through a small molecular inhibitor.

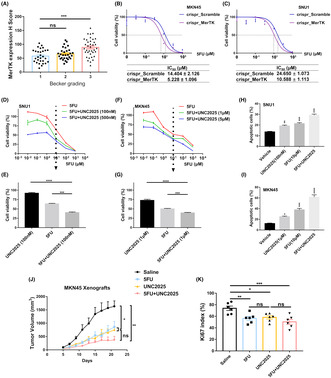

## 1 INTRODUCTION

Gastric cancer (GC) is still the fifth most common cancer, despite a gradual decrease in its incidence and mortality over the past decades.^1^ As the fourth most common cause of cancer‐related deaths, with more than 769,000 cases worldwide, the prognosis of GC remains poor, especially in advanced tumor stages. GC is divided into the following histological subtypes: adenocarcinoma, lymphomas, carcinoids, or stromal tumors. Gastric adenocarcinoma displays the majority of all cases with over 95%.^1^, ^2^ Currently, the standard treatment for advanced gastric adenocarcinoma is a combination of perioperative chemotherapy and radical surgical resection.^3^, ^4^ Currently, the preferred chemotherapy regimen for gastric adenocarcinoma consists of 5‐fluorouracil (5‐FU) and oxaliplatin. It showed a higher histopathological response and improved survival compared with previously used chemotherapeutic combinations.^5^ Recently, novel targeted therapies have been introduced for solid tumors, such as inhibition of receptor tyrosine kinases (RTK).^6^ In 2010, the randomized ToGA trial was able to show that patients with gastric cancer and human epidermal growth facter receptor 2 (HER2) protein overexpression had beneficial effects of therapy consisting of chemotherapy and HER2‐specific blockade through monoclonal antibody trastuzumab^7^ One family of RTKs is the TAM receptor (TAMR) family, comprising MerTK, Axl, and Tyro3, all of which share a similar structure. The human *MerTK* gene is located on chromosome 2q14.1 and was first cloned as a proto‐oncogene from a B lymphoblastoid cell line.^6^ Aberrant MerTK expression has been detected in hematological malignancies such as acute lymphoblastic leukemia (ALL) and acute myeloid leukemia (AML).^6^, ^8^ In solid tumors, ectopic MerTK overexpression was found in 69% of nonsmall‐cell lung cancers (NSCLC) and 70% of breast carcinomas.^9^, ^10^, ^11^ Moreover, it is highly expressed in astrocytoma, schwannoma, and melanoma.^12^, ^13^, ^14^ Structure‐based pyrazolopyrimidine UNC2025 has MerTK‐ and FLT3‐blockage activity and exhibits high selectivity for MerTK compared with other TAMR.^15^ Therefore, it has already been applied in preclinical development for AML, ALL, NSCLC, and melanoma.^16^, ^17^, ^18^, ^19^ Newer findings indicate that the inhibition of MerTK in combination with radiation and anti‐programmed death‐1 inhibition improves the survival rates of NSCLC patients by altering the adaptive immune response.^19^

Furthermore, a treatment with the small molecule inhibitor UNC2025 of MerTK led to apoptosis in glioblastoma cell lines indicating that Mer inhibition alone may be of therapeutic value in solid tumors.^20^

Nevertheless, in gastrointestinal carcinomas, such as GC and colorectal cancer (CRC), patients with MerTK overexpression show a poor prognosis,^21^, ^22^ while the underlying mechanism is still poorly studied.^22^ As specific MerTK inhibitors, such as UNC2025, could improve future treatment options, the relevance of MerTK in GC needs further investigation.

Therefore, the aim of this study was to analyze the relevance of MerTK in gastric adenocarcinoma and evaluate the possible therapeutic benefits of the MerTK inhibitor UNC2025 in vitro and in vivo.

## 2 METHODS

### 2.1 Cell culture

The human gastric cancer cell lines MKN45 (RRID:CVCL_0434) and AGS (RRID:CVCL_0139) were purchased from ATCC®. SNU1, (RRID:CVCL_0099), SNU5, (RRID:CVCL_0078), KATO‐III, (RRID:CVCL_0371), and NCI‐N87 (RRID:CVCL_1603) were provided in collaboration with Dr. Stange, University Hospital, Carl Gustav Caruz, Dresden. MKN45, SNU1, and NCI‐N87 cells were maintained in RPMI‐1640 medium with 10% fetal bovine serum (FBS), SNU5, and KATO‐III cells in Iscove's Modified Dulbecco's medium (IMDM) supplemented with 20% FBS, and AGS cells in Ham's F‐12 medium with 10% FBS. The antibiotic penicillin/streptomycin was added to the culture medium at a final concentration of 1 U/mL. All cell lines were cultured in a 37°C humidified incubator supplied with 5% CO_2_. All experiments were performed with mycoplasma‐free cells confirmed with regular testing.

### 2.2 Patients

A total of 140 human gastric adenocarcinoma diagnosed between January 2013 and December 2015 were obtained from the Department of General, Visceral, and Transplantation Surgery, University Hospital of Heidelberg (Table S1). All patients were pathologically confirmed to have stomach adenocarcinoma or adenocarcinoma of the esophagogastric junction (AEG) and underwent curative surgical resection.

### 2.3 Immunohistochemistry staining and semi‐quantification

Immunohistochemistry (IHC) staining was performed as previously described.^19^ The antibodies used are listed in Table S2.

Semi‐quantification of MerTK and cleaved caspase‐3 was calculated using the H‐Score, which multiplies the percentage of positively stained cells with the staining intensity. Briefly, 10 high‐power fields were randomly selected for each patient. Tumor cells were counted, and cell staining intensity was defined as 0 negative, 1+ weak intensity, 2+ moderate intensity, and 3+ strong intensity. Each patient sample had an H‐score between 0 and 300 using the following equation: H‐Score = [(% of weak staining) × 1 + (% of moderate staining) × 2 + (% of strong staining) × 3]. MerTK scoring was performed blinded to clinicopathological characteristics. The Ki67 index was calculated as the ratio of positively stained cells to all tumor cells (%).

### 2.4 Protein extraction and Western blotting

Cells were seeded in 60 mm cell culture dishes and incubated for a certain period of time. The cells were lysed with 300 μL ice‐cold RIPR buffer (Thermo Fisher Scientific) containing proteinase and phosphatase inhibitors (Roche). The protein concentration was determined using a Pierce™ BCA Protein Assay Kit (Thermo Fisher Scientific). Twenty micrograms of protein was administered in a final volume of 20 μL (containing 5 μL 4× Laemmli sample buffer) and heated at 95°C for 5 min before electrophoresis.

Samples were resolved by precast 4%–12% gels in 1×TGS running buffer (Bio‐Rad). Proteins were then transferred to a nitrocellulose membrane and blocked with 5% BSA buffer for 1 h at room temperature. The membranes were incubated with primary antibodies overnight at 4°C (Table S3). The next day, the membrane was washed thrice with TBST buffer and incubated with HRP‐conjugated secondary antibodies. Target proteins were visualized by chemiluminescence using an HRP substrate (Thermo Fisher Scientific), and images were captured using a Gel Imaging and Documentation System (VilberLourmat).

### 2.5 Genetically knockdown using siRNA

Small‐interfering RNA (siRNA) targeting human MerTK was purchased from Invitrogen (HSS173654). The sequence was 5′‐GCC GCA UUG CUA AGA UGG CUG UUA A‐3′. The cells were seeded in six‐well plates at a density of 1 × 10^6^ cells/well. MerTK and negative control siRNA were transfected into cells using Lipofectamine RNAiMAX (Thermo Fisher Scientific) according to the manufacturer's guidelines. Briefly, 9 μL of Lipofectamine RNAiMAX reagent and 3 μL of siRNA stock (10 μM) were dissolved in 150 μL of Opti‐MEM (Thermo Fisher Scientific). After mixing and incubating for 5 min at room temperature, 250 μL of the mixture was added to each well. Subsequent experiments were performed after incubation for 48 h.

### 2.6 Genetically knockout using CRISPR/Cas9 systems

#### 2.6.1 CRISPR/Cas9‐mediated knockout in MKN45 and SNU1

The CRISPR/Cas9 gene editing system was used to generate a stable MerTK knockout phenotype in the MKN45 and SNU1 cell lines. Single guide RNA (sgRNA) targeting MerTK was designed using the database on the CRISPOR website (Table S4). LentiCRISPRv2 (Addgene) containing Cas9 was used as the vector for sgRNA and successful construction was confirmed by Sanger Sequencing. Next, the modified plasmids were transfected into HEK‐293T cells using polyethylenimine to obtain *MerTK*‐lentiCRISPRv2 lentivirus. Viral particles were harvested after 3 days and centrifuged at 131,000 ***g*** for 3 h. MKN45 and SNU1 cells were transfected with the lentivirus and incubated for 48 h. Selection was performed by adding 10 μg/mL puromycin (InvivoGen) to the culture medium. Cells were seeded in 96 well plates at a density of one cell per well to obtain single clones. Once single cells grew into colonies, they were transferred to 24 well plates for further culture until sufficient for DNA sequencing and protein extraction. Knockout of MerTK was verified using Sanger Sequencing and western blotting.

#### 2.6.2 CRISPR/Cas9‐mediated knock out in SNU5

The lentiviral Cas9‐containing plasmid lentiCRISPR v2 (Ref.^23^ was a gift from F. Zhang) (Addgene #52961, Cambridge, MA, USA). gRNAs for *MERTK* were designed using CrispRGold program version 1.1 (https://crisprgold.mdc‐berlin.de). Cloning of the gRNA into the lentiCRISPR v2 was performed according to the Zhang lab protocol. Lentiviral packaging and transduction were performed as described.^24^ Cells were supplemented with Puromycin for 7 days to select positive clones.

### 2.7 Cell proliferation assay

Cell proliferation assay was performed using a BrdU colorimetric ELISA kit (Sigma‐Aldrich). Cells were seeded in 96‐well plates at a density of 5 × 10^3^/well and incubated for 24 h in a 37°C incubator. The next day, 10 μL of BrdU labeling solution was added to each well and incubated for 2 h. Then, the culture medium was removed, and the cells were fixed with a fixation buffer for 30 min. The anti‐BrdU‐POD solution (100 μL) was added to each well and incubated for another 2 h. The cells were then washed three times with PBS, and 100 μL substrate buffer was added to each well. When the color development was sufficient, 25 μL of 2 M HCL was added to stop the reaction. Absorbance was measured at 450 nm using an ELISA plate reader.

### 2.8 Cell viability assay

Cell viability was tested using the WST‐1 reagent. The compounds were dissolved in DMSO to make a 10 mM stock solution for UNC2025 and a 500 mM stock solution for 5‐FU. Cells were seeded in 96‐well plates at a density of 5 × 10^3^ cells/well and incubated with different concentrations of the test compounds for 48 h. Ten microliters of WST‐1 reagent was added to each well and incubated for 2–4 h. When color development was sufficient, absorbance was measured using an ELISA reader at 450 nm.

### 2.9 Soft‐agar colony formation assay

Agar (1% w/v) was melted in the culture medium, and 2 mL agar solution was transferred to cover the six‐well plates for each well. Once the bottom layer of agar solidified, 1.5 mL of 0.6% w/v low‐melting agarose in a culture medium containing 1 × 10^3^ cells was added to each well. Fresh medium (100 μL) was added every 3 days per well to prevent desiccation. The six‐well plates were incubated in a cell culture incubator for 2–3 weeks. Colonies were stained and counted by adding 200 μL nitroblue tetrazolium chloride solution to each well and incubating overnight.

### 2.10 Flow cytometry

Cell cycle analysis was performed using propidium iodide (PI) staining to determine the DNA content. Cells (1 × 10^6^) were seeded in six‐well plates and treated with UNC2025 for 48 h. The cells were fixed in 70% ethanol overnight with 50 μg/mL propidium iodide (PI) and 25 μg/mL RNase. The next day, the cells were washed twice with PBS and resuspended for flow cytometry (FACSuite, BD). The percentage of cells in the G1, G0/G1, S, and G2/M phases, and cells with polyploidy were calculated.

Apoptosis was analyzed by Annexin V/PI staining. Cells were treated with UNC2025 for 48 h and resuspended in 1× Annexin V binding buffer at a density of 1 × 10^6^ cells/mL. The cell suspension (100 μL) was transferred to an FACS tube with 5 μL Annexin V and 5 μL PI and incubated at room temperature for 15 min in the dark. Then, 400 μL binding buffer was added to each sample, and flow cytometric measurement was performed within 1 h (FACSuite, BD).

### 2.11 Murine gastric cancer xenograft model

All animal experiments were approved by the Animal Welfare Office of Baden‐Wuerttemberg, Germany, and were performed under protocol G94/20. Female athymic nude mice, NU(NCR)‐Foxn1^nu^ (4–6 weeks), were purchased from Charles River. The human gastric cancer cell line MKN45 (1 × 10^6^ cells, wild‐type and CRISPR/Cas9‐modified) was suspended in 100 μL culture medium containing 50% Matrigel and injected subcutaneously into the right flank. Tumor volumes were measured using an external caliper every other day and calculated using the formula [(width × width× length)/2]. Once the average tumor volume reached 100 mm^3^, the mice were randomly grouped. UNC2025 was administered by oral gavage at 10 mL/kg once daily until the end of the experiment. 5FU was injected intraperitoneally for three continuous days per week at 30 mg/kg for 2 weeks. Mice were sacrificed at the endpoint and tumors were extracted for further analysis.

### 2.12 Statistics

Statistical analysis was performed using GraphPad Prism 7.0 (GraphPad Software). The correlation between the MerTK H‐Score and clinicopathological characteristics was analyzed using the Mann–Whitney *U* test and the Kruskal–Wallis test. The Kaplan–Meier method and log‐rank test were used to perform the survival analysis. Student's *t*‐test was performed to compare the differences between the two groups. Differences between the tumor growth curves were analyzed using two‐way ANOVA. All in vitro experiments were repeated at least three times, and the representative results are shown. Data are presented as the mean ± SEM. Statistical significance was defined as *p*‐value <0.05.

## 3 RESULTS

### 3.1 MerTK high expression reveals poor prognosis in human gastric adenocarcinoma

**FIGURE 1:**
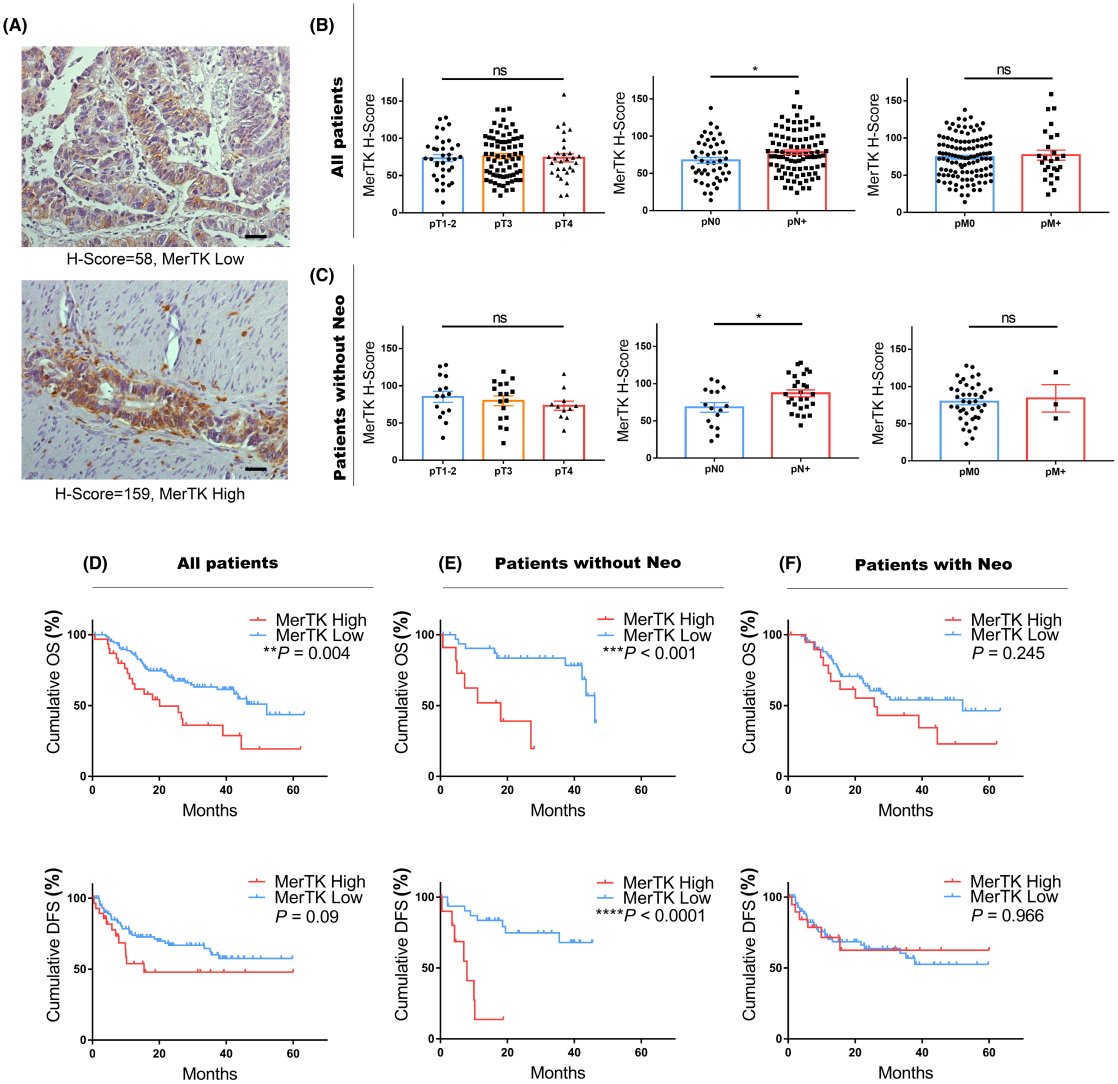
MerTK expression in human gastric adenocarcinoma. (A) Representative images of MerTK staining in human gastric adenocarcinoma samples. Plots of MerTK H‐Score showed no correlation with pathological T (B, C) and M (B, C) stages (the Kruskal–Wallis test, ns, not significant) but positively correlated with lymph node metastasis (B, C) for all patients (*n* = 140) and patients without preoperative chemotherapy (*n* = 44) (the Mann–Whitney test, *p < 0.05). Kaplan–Meier analysis of OS and DFS was performed in connection with MerTK expression for all patients (D; *n* = 140), patients without neoadjuvant chemotherapy (E; *n* = 44), and patients who underwent preoperative chemotherapy (F; *n* = 96). Statistical significance was determined by the log‐rank test. Scale bars, (A): 50 μm.

To examine the expression level of MerTK in human gastric adenocarcinoma, we selected 140 patients diagnosed with pathologically confirmed gastric adenocarcinoma. The clinicopathological characteristics of all the patients are listed in Table S1. Histological evaluation of MerTK was performed using IHC staining (Figure 1A). Generally, a moderate level of MerTK staining was observed, with a median H‐score of 75 (range: 14–159) in the analyzed cohort. To further explore the effect of high MerTK expression on the prognosis of patients with gastric cancer, we defined an H‐score of 98 (mean value +1 SEM) as the cutoff value, with an H‐Score equal to or above 98 considered as MerTK high expression, while under 98 was determined as MerTK low expression. As a result, 31 patients (22.14%) showed high MerTK expression. MerTK expression was not significantly associated with age, sex, grading, tumor location, preoperative therapy status, or clinical stages (cT, cN, and cM) (Figure S1). Although there was no difference between MerTK status and pathological T or M stage (Figure 1B), there was a positive correlation between MerTK expression and pathological lymph node metastasis. Patients with a high MerTK expression status had a higher risk of lymph node metastasis in the postoperative specimens. This correlation remained in the subgroup of 44 patients who did not receive neoadjuvant chemotherapy (Figure 1C). For the entire cohort, patients with high levels of MerTK had shorter overall survival (OS) but not disease‐free survival (DFS) (Figure 1D). Interestingly, for 44 patients who did not receive neoadjuvant chemotherapy, the MerTK high group exhibited a poorer prognosis than the MerTK low group, both for OS and DFS (Figure 1E). In contrast, for patients who underwent neoadjuvant chemotherapy, there was no difference in survival between the two groups (Figure 1F). Subsequent multivariate Cox regression analysis showed that high MerTK expression was an independent OS factor for gastric cancer (HR = 2.250, 95% CI = 1.301–3.891, *p* < 0.05) (Table 1).

**TABLE 1:** Univariate and multivariate COX regression analyses for overall survival (OS) in gastric adenocarcinoma patients.

Variables	Univariate analysis				Multivariate analysis			
	HR	95% CI		*p* Value	HR	95% CI		*p* Value
MerTK expression	2.250	1.301	3.891	0.004	2.074	1.167	3.688	0.013
Age	1.037	0.600	1.790	0.898	NA			
Gender	0.772	0.429	1.389	0.388	NA			
Neoadjuvant Chemotherapy	1.193	0.670	2.125	0.548	NA			
Pathological T stage								
T1	1.838	0.355	9.511	0.468	NA			
T2	1.115	0.240	5.184	0.890	NA			
T3	1.311	0.315	5.448	0.710	NA			
T4	1.206	0.242	6.009	0.819	NA			
Pathological N stage								
N1	1.695	0.711	4.044	0.234	NA			
N2	2.410	1.058	5.491	0.036	1.816	0.765	4.309	0.176
N3	3.548	1.790	7.033	<0.001	3.406	1.660	6.990	0.001
Pathological M stage	2.760	1.572	4.845	<0.001	2.215	1.240	3.956	0.007

Abbreviations: CI, confidence interval; HR, hazard ratio; NA, not applicable.

### 3.2 MerTK facilitates proliferation in gastric adenocarcinoma in vitro

**FIGURE 2:**
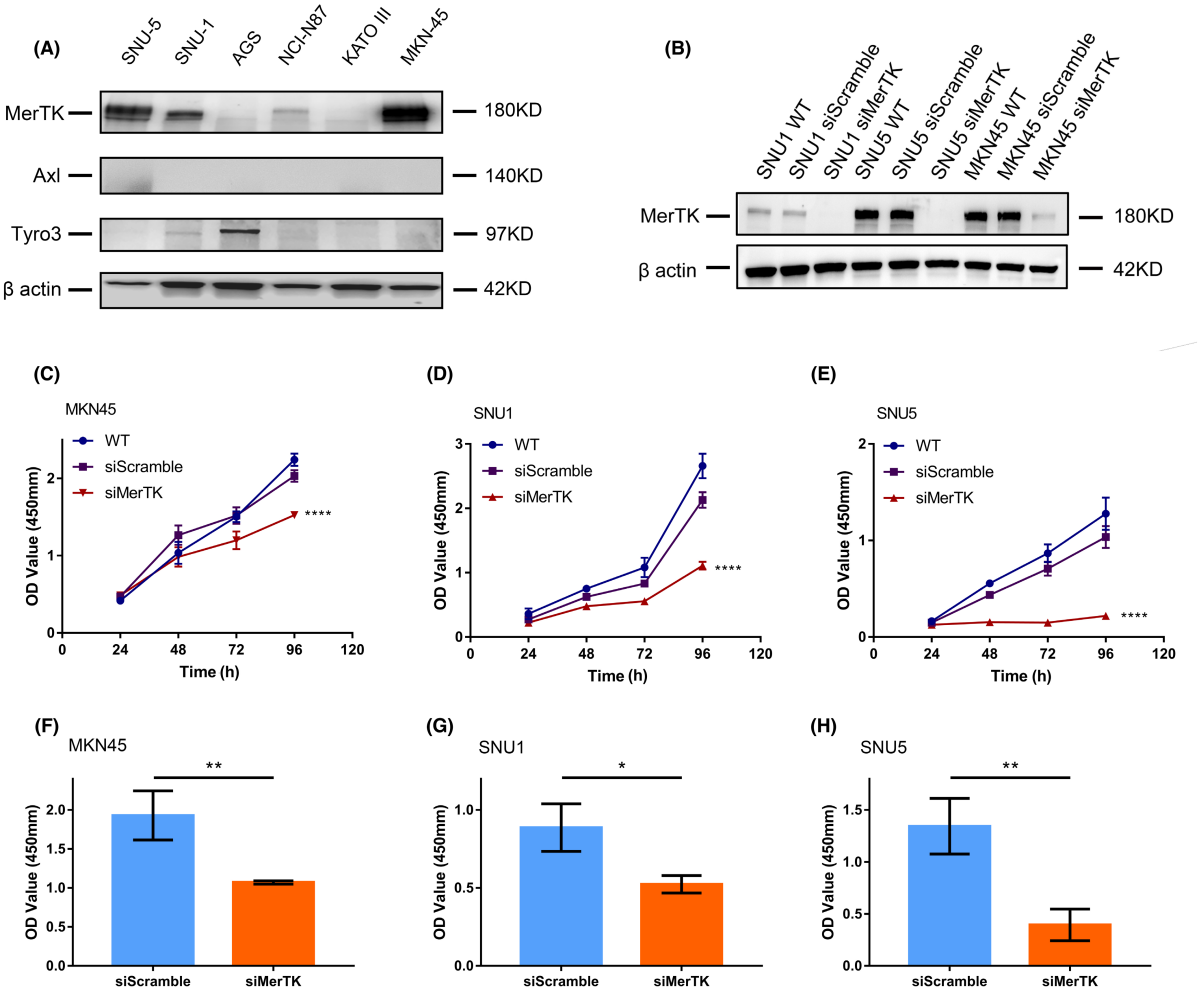
MerTK facilitates proliferation in gastric adenocarcinoma cells. (A) Representative image of western blot for MerTK, Axl, and Tyro3 in gastric adenocarcinoma cell lines. (B) Reduced expression level of MerTK was detected after transfected with siRNA targeting MerTK. (C–E), WST‐1 assay showed cell viability was significantly decreased in SNU1, SNU5, and MKN45 cells after MerTK knockdown (two‐way ANOVA, ****p < 0.0001). (F–H) ELISA assay was performed with 2 h incubation of BrdU in SNU1, SNU5, and MKN45 after transfected with siRNA for 48 h. Results showed knockdown of MerTK inhibited the synthesis level of DNA compared with the negative control (Student's *t*‐test, *p < 0.05; **p < 0.01).

To explore the role of MerTK in gastric adenocarcinoma (GAC), we analyzed the expression of MerTK and other TAMR family members Axl and Tyro3 in a panel of gastric cancer cell lines (MKN45, SNU1, SNU5, KATO‐III, NCI‐N87, and AGS). Of the six cell lines, MKN45, SNU5, and SNU1 had detectable expression of MerTK, with Tyro3 only detected in AGS, and none of the cell lines expressed Axl (Figure 2A). To analyze the function of MerTK, knockdown was performed in the SNU5, SNU1, and MKN45 cell lines with transfection of siRNA targeting MerTK (Figure 2B). Tumor cell proliferation decreased in all three cell lines with MerTK knockdown (MerTK KD) groups compared with the control and wild‐type groups (Figure 2C–E). Consistent with the WST‐1 results, which are metabolism‐based, a decreased level of DNA synthesis was observed after MerTK KD as measured by BrdU assays, confirming that MerTK promotes the proliferation of GAC in vitro (Figure 2F–H).

### 3.3 Genetic MerTK knockout inhibits colony formation

**FIGURE 3:**
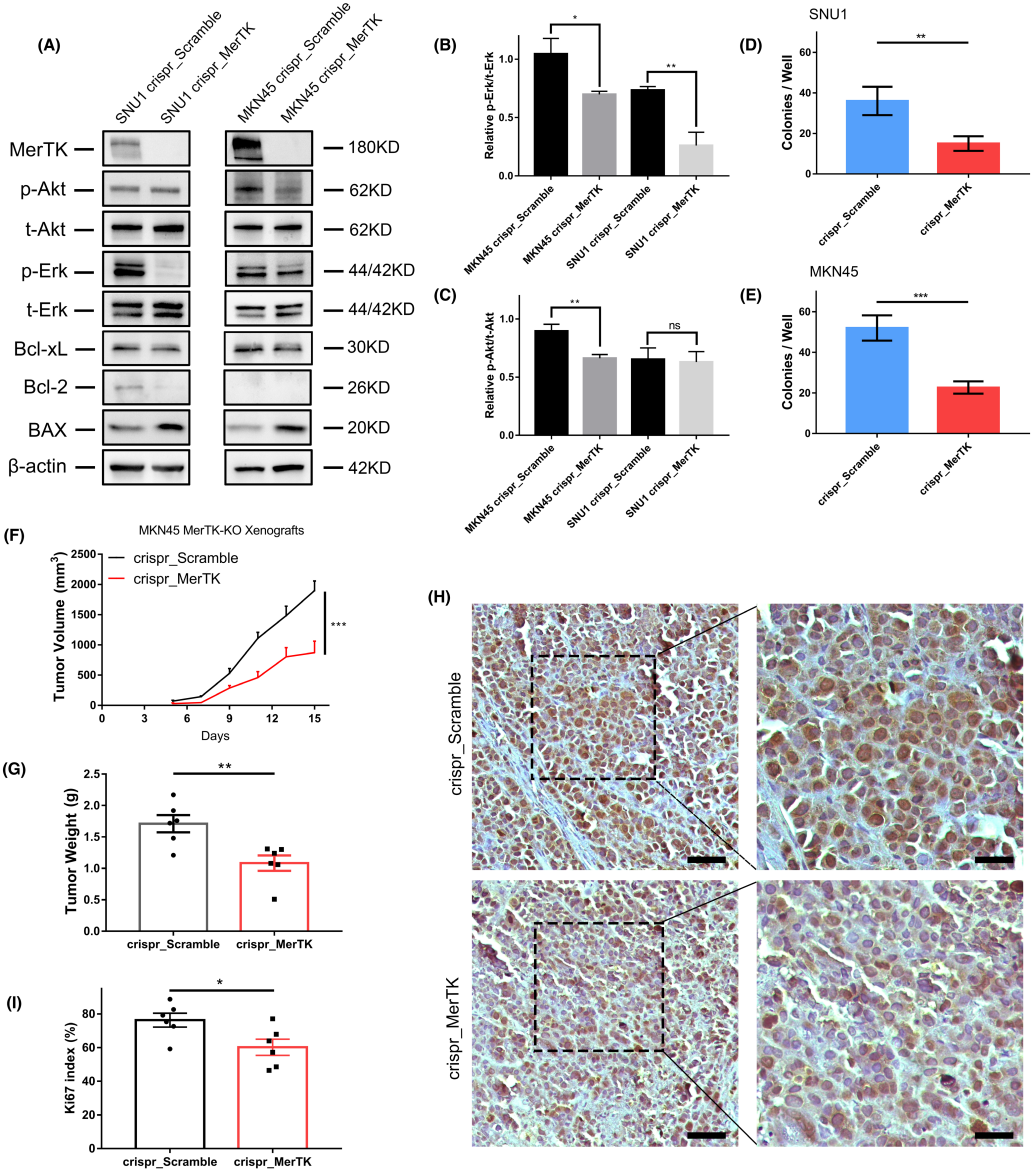
MerTK knockout dampens tumor growth both in vitro and in vivo. (A) Western blot showed the absence of MerTK after genetic knockout using CRISPR/Cas9 system. Alterations of downstream signaling factors were detected and a representive image was presented. (B, C) Histogram of relative expression of p‐Erk/t‐Erk and p‐Akt/t‐Akt after MerTK knockout. (D, E) Histogram showed different number of colonies between crispr_Scramble and crispr_MerTK groups in SUN1 and MKN45 cells. (F) Tumor growth of MKN45 crispr_MerTK and crispr_Scramble derived xenografts. Both reduced tumor volumes (F) and tumor weights (G) were observed in the crispr_MerTK group compared to the control 24 groups; (*n* = 6 per group, two‐way ANOVA, ***p < 0.001). (H) Representative images of histological evaluation of the Ki67 index via IHC staining. Scale bar: left, 50 μm; right, 25 μm. (I) Histogram showing a lower Ki67 index in crispr_MerTK group. For (B–E, G, and I) Student's *t*‐test, *p < 0.05, **p < 0.01, ***p < 0.001, ns, not significant. Scale bars: (H), left, 50 μm; right, 25 μm.

To establish a stable MerTK knockout in SNU1 and MKN45 cells, we used the CRISPR/Cas9 system, and the phenotype was confirmed by DNA sequencing and protein levels (Figure 3A and Figure S2). To investigate possible altered downstream targets, especially in proliferative and anti‐apoptotic signaling pathways after knockout of MerTK in SNU1 and MKN45 cells, western blot analysis was performed including the MAPK and AKT pathways and revealed a reduction in the phosphorylation of Erk, which is considered a key regulator of the cell cycle (Figure 3A,B). Decreased phosphorylation of Akt was detected only in MKN45 cells, but not in SNU1 cells, indicating that cells may overcome and bypass this pathway (Figure 3A,C). The anti‐apoptotic marker Bcl‐xL was downregulated after MerTK‐KO in both cell lines and Bcl‐2 was downregulated in SNU1 but undetectable in MKN45 cells; however, the level of pro‐apoptotic BAX was upregulated in both cell lines (Figure 3A). These results demonstrate that MerTK may play a role in the apoptosis of gastric adenocarcinoma cells. The effect of MerTK on anchorage‐independent growth was assessed using a soft agar colony formation assay. A robust decrease in the number of colonies in the MerTK‐KO groups compared to that in the control groups was detected, indicating that MerTK may be important for GC cells to form colonies from a single cell (Figure 3D,E).

### 3.4 Knockout of MerTK dampens tumor growth in a mouse model of gastric adenocarcinoma

To examine the effect of MerTK on tumor progression in vivo, a xenograft mouse model of GAC was established. MerTK‐KO and negative control MKN45 cells were injected subcutaneously into athymic nude mice, and tumor volumes were measured every other day. Compared with the control group, tumor volumes and weights were significantly decreased after MerTK‐KO (Figure 3F,G). IHC staining revealed a reduced level of proliferation in the MerTK‐KO group compared to that in the control group, indicating an important role for MerTK in tumor growth in vivo (Figure 3H,I).

### 3.5 UNC2025, a small molecule inhibitor, induces cell‐cycle arrest and apoptosis in GAC

**FIGURE 4:**
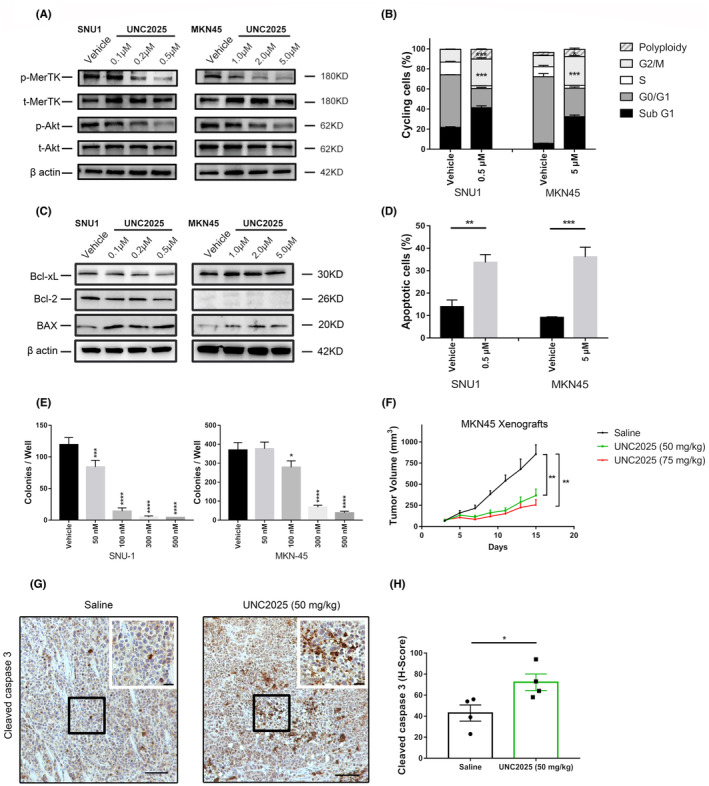
UNC2025 induces apoptosis, inhibits proliferation in gastric adenocarcinoma, and shows therapeutic effect in vivo when used alone. (A) Western blot showed a dose‐dependent decrease in the phosphorylation level of MerTK and Akt after treatment with UNC2025 for 3 h in MKN45 and SNU1 cells. (B) Histogram showed the percentage of cells in the Sub G1, G0/G1, S, G2/M phases and cells with polyploidy after being treated with UNC2025 for 48 h. (C) Representative image of western blot targeting Bcl‐2, BclxL, and BAX after being treated with different concentrations of UNC2025 for 24 h in SNU1 and MKN45 cells. (D) Flow cytometry showed an increased proportion of early and late apoptotic cells (Annexin V positive) after being treated with UNC2025 for 48 h. (E) SNU1 and MKN45 cells were cultured in soft agar and overlaid with a medium containing different concentrations of UNC2025. After cultured for 3 weeks, a significant reduction of the colony numbers were 26 observed in the UNC2025 groups. (F) UNC2025 was administrated by oral gavage daily at a concentration of 50 and 75 mg/kg in MKN45‐derived xenografts. Saline vehicle was given at 10 mL/kg as a control. Reduced tumor volumes were detected after treatment with UNC2025 (*n* = 4 per group, two‐way ANOVA, **p < 0.01). (G) Representative images of cleaved caspase‐3 staining in Saline group and UNC2025 (50 mg/kg) group. Scale bars: overview, 100 μm; inset, 50 μm. (H) Plots of H‐Score of cleaved caspase‐3 in two groups. For (B) and (E), one‐way ANOVA, *p < 0.05, ***p < 0.001, ****p < 0.0001. For (D) and (H), Student's *t*‐test, *p < 0.05, **p < 0.01, ***p < 0.001.

To investigate whether MerTK inhibition has therapeutic value in GC, we used UNC2025 as a specific inhibitor targeting MerTK. In vitro treatment with different concentrations of UNC2025 for 3 h resulted in a significant dose‐dependent decrease in the phosphorylation of MerTK and Akt in SNU1 and MKN45 cells (Figure 4A). The IC_50_ of UNC2025 in different cell lines was tested using the WST‐1 assay (Figure S3A). Flow cytometry showed a decrease in the number of cells in the G0/G1 phase upon UNC2025 treatment (IC_50_ dose) for 48 h, while the percentage of cells in the G2/M phase and cells with polyploidy significantly increased (Figure 4B and Figure S3B). Consistently, UNC2025‐treated cells showed an increased level of BAX compared with the vehicle control group (Figure 4C), and flow cytometry using Annexin V/PI staining further confirmed the increased ratio of apoptotic cells after UNC2025 treatment (Figure 4D and Figure S3C,D). Taken together, these findings suggest that MerTK inhibition induces apoptosis of GAC cells. In accordance with MerTK‐KO, MerTK inhibition using UNC2025 resulted in a dose‐dependent decrease in the number of colonies on soft agar (Figure 4E). Interestingly, even at lower concentrations of UNC2025, colony formation was significantly reduced in both cell lines, indicating an important role of MerTK in anchorage‐independent growth.

We further examined the efficiency of MerTK inhibition by UNC2025 in vivo. Wild‐type MKN45 cells were injected subcutaneously to establish a xenograft gastric cancer model. A significant reduction in tumor volume was observed in the UNC2025 treatment groups compared to that in the control group (Figure 4F). In addition to the in vitro findings, a higher H‐score of cleaved caspase‐3 was detected with IHC staining, demonstrating that MerTK inhibition induced apoptosis and therefore represents a potent therapeutic target for GAC (Figure 4G,H).

### 3.6 MerTK high expression attenuates the response toward 5‐FU‐based neoadjuvant chemotherapy

**FIGURE 5:**
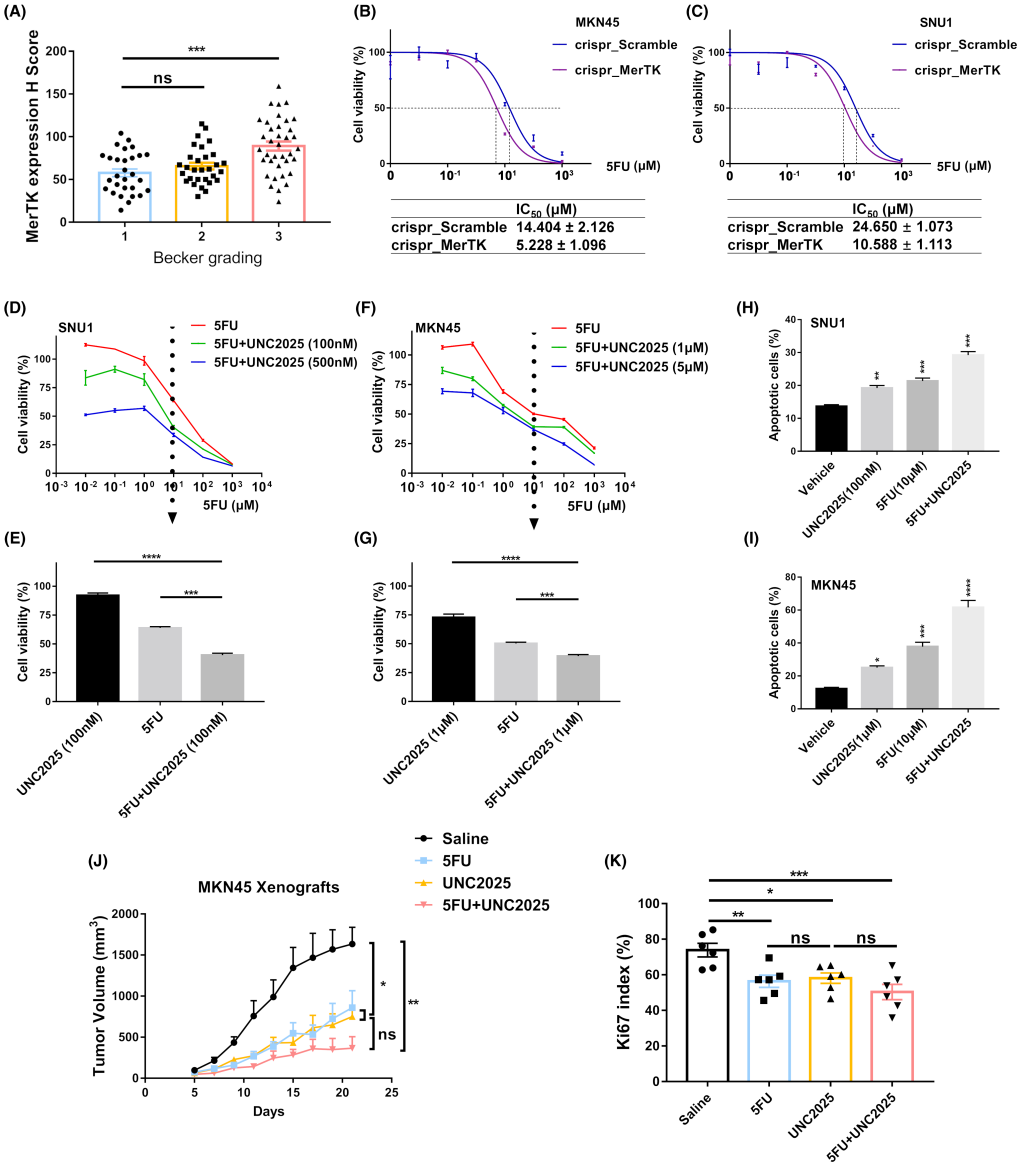
MerTK inhibition sensitizing 5FU‐based chemotherapy in gastric adenocarcinoma via inducing apoptosis. (A) Plots of the MerTK H‐Score in Becker grading system revealed MerTK overexpression is highly associated with Becker grade 3 (the Kruskal–Wallis test, ***p < 0.001, ns, not significant). (B and C) CRISPR/Cas9‐modified cells were seeded in 96‐well plates and treated with different concentrations of 5FU for 48 h. Cell viability was assessed by the WST‐1 assay and the IC50 was calculated for each group. (D, F) SNU1 or MKN45 cells were treated with different concentrations of 5FU or 5FU plus UNC2025 for 48 h. Cell viability was assessed by the WST28 1 assay. (E, G) Histogram showed the status of cell viability at 10 μM. A relatively small dosage of UNC2025 could enhance the antitumor effect of 5FU. (H, I), Flow cytometry showed an increased level of apoptotic cells (Annexin V positive) when combined 5FU with UNC2025. (J) MKN45‐derived xenografts were divided into four groups, saline control (10 mL/kg), 5FU (30 mg/kg), UNC2025 (50 mg/kg), and the combination group of 5FU and UNC2025. Tumor growth curves were shown (*n* = 6 per group, two‐way ANOVA, *p < 0.05, **p < 0.01). (K) Histogram showed different Ki67 indices between groups via IHC staining. For (E, G, H, I, and K), one‐way ANOVA, *p < 0.05, **p < 0.01, ***p < 0.001, ****p < 0.0001.

In the present cohort, no MerTK‐dependent survival difference was observed in patients who underwent neoadjuvant chemotherapy (Figure 1L,M), and patients with a high expression of MerTK were more frequently nonresponders to neoadjuvant therapy (Becker response grade 3), indicating that high MerTK expression may attenuate the anti‐tumor effect of chemotherapeutic agents (Figure 5A). 5‐Fluorouracil (5FU) is one of the elements of the FLOT regimen, which is now the standard modality for neoadjuvant chemotherapy. To investigate whether MerTK mediates 5‐FU resistance in gastric cancer cells, the IC_50_ of 5‐FU was evaluated. Compared with the control groups, the absence of MerTK in knockout groups sensitized the antitumor effect both in MKN45 and SNU1 cells, reducing the IC_50_ by more than twofold (Figure 5B,C). A similar effect was observed in wild‐type cells under UNC2025 treatment at low and high concentrations in combination with 5FU, oxaliplatin, and docetaxel. Cell viability was tested using the WST‐1 assay, and the results revealed that even at a lower concentration, UNC2025 could enhance the therapeutic effect of 5FU but not oxaliplatin or docetaxel (Figure 5D,E and Figure S4). Flow cytometry revealed a larger proportion of apoptotic cells after treatment with the combination of 5FU and UNC2025 (Figure 5H,I and Figure S5), indicating that MerTK inhibition could enhance the antitumor effect of 5‐FU. These results are supported by in vivo experiments on MerTK inhibition in combination with chemotherapy. 5FU combined with UNC2025 exhibited a more robust antitumor effect in comparison with the control without a significant difference between the treatment groups. Nonetheless, the combination of 5FU and UNC2025 showed a higher reduction than 5FU or UNC2025 alone in contrast to the control in tumor growth with a significantly decreased Ki67 index. Still, there were no significant differences in direct comparison of the treatment groups in regard to the Ki67 index (Figure 5J,K).

## 4 DISCUSSION

Here, we showed the relevance of MerTK expression as an independent factor associated with overall survival in human gastric adenocarcinoma in addition to histopathological M status using multivariant Cox regression analysis. Furthermore, we showed that the inhibition of MerTK using knockdown or knockout strategies was followed by reduced cell viability, likely through a higher rate of apoptosis and, consequently, smaller tumor growth in vivo. Similar effects were observed using the MerTK inhibitor UNC2025 in vitro and in vivo, making MerTK a potential new target for future therapies.

The inhibition of MerTK, as well as other TAMR members, has been developed in many preclinical investigations.^12^, ^16^, ^18^, ^22^, ^25^ In GC, a previously conducted knockdown of MerTK using small hairpin RNA (shRNA) showed decreased proliferation compared to the control.^13^ A pan‐TAMR inhibitor, RXDX‐106, reduces cell viability in MerTK‐positive GC cell lines and patient‐derived cell lines.^26^ However, neither the effect nor the mechanism of MerTK‐specific inhibition in GC has been previously identified. In the present study, we inhibited MerTK in different ways by siRNA knockdown, genetic knockout via the CRISPR/Cas9 system, and specific blockage using UNC2025. Our results indicate that MerTK inhibition leads to a significant decrease in the proliferation and colony formation capabilities of soft agar. Interestingly, the viability of suspended tumor cells was more vulnerable to MerTK inhibition, indicating that MerTK plays a vital role in maintaining cell viability in an anchorage‐independent manner. Malignant cells have to overcome anoikis when growing in a new microenvironment different from the primary site, and this is considered the first step in the metastasis of tumor cells.^27^, ^28^ Our results showed a decreased number of soft agar colonies after MerTK dysfunction, which may explain why patients with high MerTK expression were more likely to have lymph node metastasis.

A previous study on glioblastoma showed that knockdown of MerTK disrupted the rounded morphology of glioma cells with a decrease in tumor stem cell markers, such as Sox2 and Nestin, indicating that MerTK could maintain cells in an undifferentiated state.^29^ Given that peritoneal cavity metastasis is one of the major factors affecting the prognosis of GC patients,^30^ MerTK inhibition would be of great value in GAC therapy, decreasing the number of anchorage‐independent cells and thereby reducing the probability of metastasis.

Neoadjuvant chemotherapy is now the standard approach for patients with advanced gastric adenocarcinoma^2^; however, not all patients benefit from this modality. Developing an effective prediction system before initiating neoadjuvant chemotherapy is a great challenge to avoid the side effects of chemotherapeutic drugs in nonresponding patients. Chemoresistance mediated by MerTK has been detected in many types of cancer.^31^ In our cohort, 69% of all patients (*n* = 96) underwent FLOT chemotherapy before surgery. The Becker grading system was used to evaluate treatment response. Although high MerTK expression showed no survival risk in this group of patients, it demonstrated the potential ability to reflect chemotherapy response. Patients with higher MerTK H‐scores tended to show resistance to neoadjuvant chemotherapy, indicating that MerTK may be a predictive marker. Dysfunction of MerTK seemed to sensitize the antitumor effect of 5‐FU in this study. A similar effect was detected in a colorectal cancer study, in which knockdown of MerTK enhanced the antiproliferative effect of 5‐FU in HCT116 cells.^22^ This promising finding may help overcome or at least reduce chemoresistance, as 5‐FU is a major component of traditional chemotherapy regimens.

After the inhibition of MerTK with UNC2025 or MerTK knockout in MKN45 cells, a parallel decrease in Akt phosphorylation was detected. In combination with an increased apoptosis rate after treatment with UNC2025, these results suggest that MerTK induces chemoresistance by activating downstream pro‐survival signaling pathways.

In summary, high MerTK expression predicted not only poor prognosis but also an unsatisfactory response to neoadjuvant chemotherapy. Higher expression of MerTK was positively associated with lymph node metastasis in GC patients, and knockout of MerTK reduced tumor growth and colony formation capability. MerTK inhibition by UNC2025 exhibited therapeutic relevance both in cell culture and in an in vivo model of GC. Finally, MerTK inhibition seems to sensitize the cells to 5‐FU‐based chemotherapy, indicating its possible role in improving the therapeutic response. This could provide novel treatment opportunities for patients undergoing refractory neoadjuvant treatment in the future.

## 5 CONCLUSION

In this study, we demonstrated that MerTK was an independent prognostic factor for GAC and that inhibition of MerTK not only reduced tumor progression but also enhanced the effect of 5‐FU‐based chemotherapy.

## AUTHOR CONTRIBUTIONS

**Naita Maren Wirsik:** Formal analysis (equal); writing – original draft (lead); writing – review and editing (lead). **Mingyi Chen:** Investigation (lead). **Liping He:** Formal analysis (equal); investigation (equal). **Uraz Yasar:** Formal analysis (equal); investigation (equal). **Justus Gaukel:** Data curation (equal). **Alexander Quaas:** Formal analysis (equal); methodology (equal); validation (equal). **Henrik Nienhüser:** Supervision (equal); writing – review and editing (equal). **Ella Leugner:** Formal analysis (equal); investigation (equal). **Shuai Yuan:** Data curation (equal); investigation (equal). **Nikolai Schleussner:** Visualization (equal); writing – review and editing (equal). **Jin‐On Jung:** Data curation (supporting); software (equal). **Jadie Sue Plücker:** Data curation (equal); investigation (equal); validation (equal). **Martin Schneider:** Project administration (supporting); resources (equal); supervision (equal). **Thomas Schmidt:** Conceptualization (equal); methodology (equal); project administration (lead); resources (lead); supervision (lead); writing – original draft (equal); writing – review and editing (lead).

## FUNDING INFORMATION

The study was supported by an internal university project grant from the Foundation of Stiftung Chirurgie of the surgical department of the University Hospital Heidelberg. The revision was supported by the Cologne Clinician Scientist Program (CCSP)/Faculty of Medicine/University of Cologne. Funded by the German Research Foundation (DFG, FI 773/15‐1).

## CONFLICT OF INTEREST STATEMENT

The authors declare no conflict of interest.

## ETHICAL APPROVAL

This study was approved by the institutional ethics committee, and informed consent was obtained from all the patients. This study was performed with permission from the Ethics Committees of the University of Heidelberg (No: S‐635/2013).

## Supporting information


Figures S1–S5.

Table S1.

Tables S2–S4.

## Data Availability

Datasets generated and analysed for this work are not publicly available. But they are accessible upon reasonable request.
