# Supplementary material for: Targeting the receptor tyrosine kinase MerTK shows therapeutic value in gastric adenocarcinoma
Source: Cancer Med. 2024 Mar 28;13(7):e6866. doi: 10.1002/cam4.6866 (PMC10974716; doi:10.1002/cam4.6866)
Supplement: Supplementary file 1 — Figures S1–S5. [file CAM4-13-e6866-s003.dotx]

**Supplementary Figure 1**


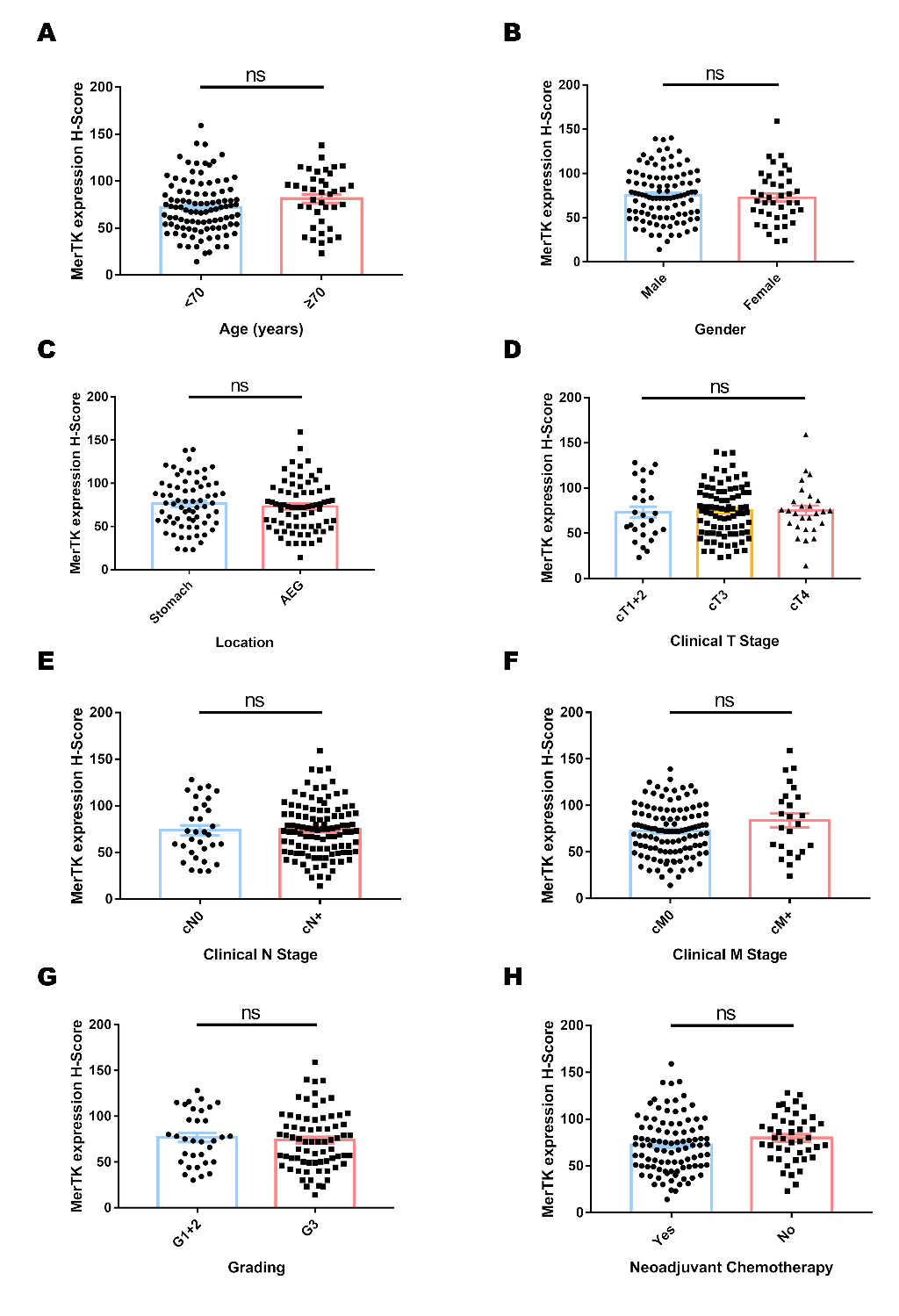


**Supplementary Figure S1:** **Correlation between the MerTK expression and clinicopathological characteristics**

Plots of MerTK H-Score showed no correlation with age (**A**), gender (**B**), location (**C**), clinical stages (**D**~**F**), grading (**G**), and the status of neoadjuvant chemotherapy (**H**). *n* = 140, the Mann-Whitney test and the Kruskal-Wallis test, ns, not significant.

**Supplementary Figure 2**


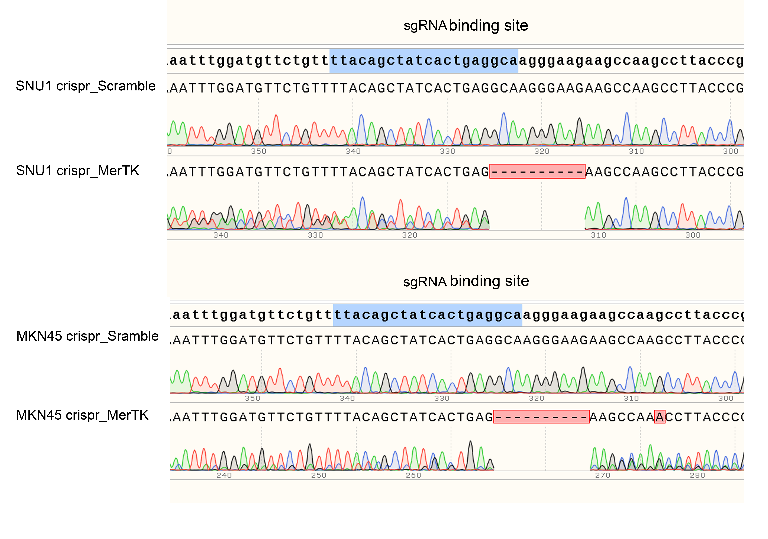


**Supplementary Figure S2:** **Sanger sequencing after MerTK-KO using CRISPR/Cas9 system**

Total DNA was extracted using DNeasy Blood & Tissue Kits (Qiagen) based on the manufacturer’s instruction. Polymerase chain reaction (PCR) was performed using *Taq* polymerase kit (Minerva Biolabs). Sanger Sequencing was performed by EuroFin company based on their protocol and revealed a 10bp deletion adjacent to the sgRNA binding site in MKN45 MerTK-KO and SNU1 MerTK-KO cell lines.

**Supplementary Figure 3**


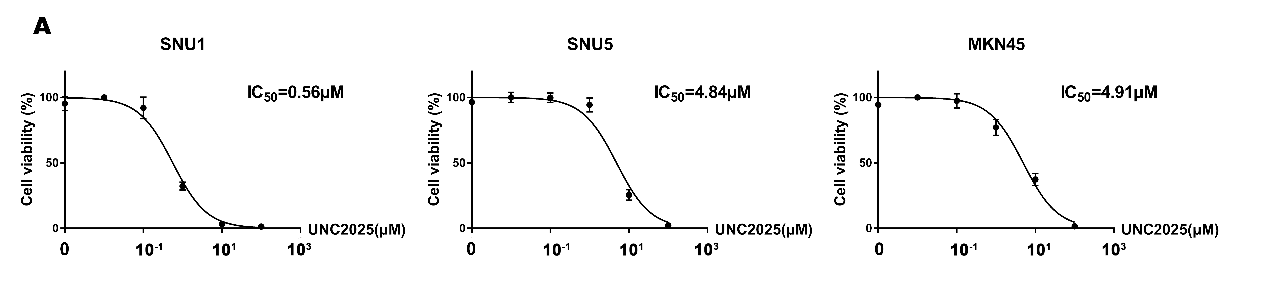


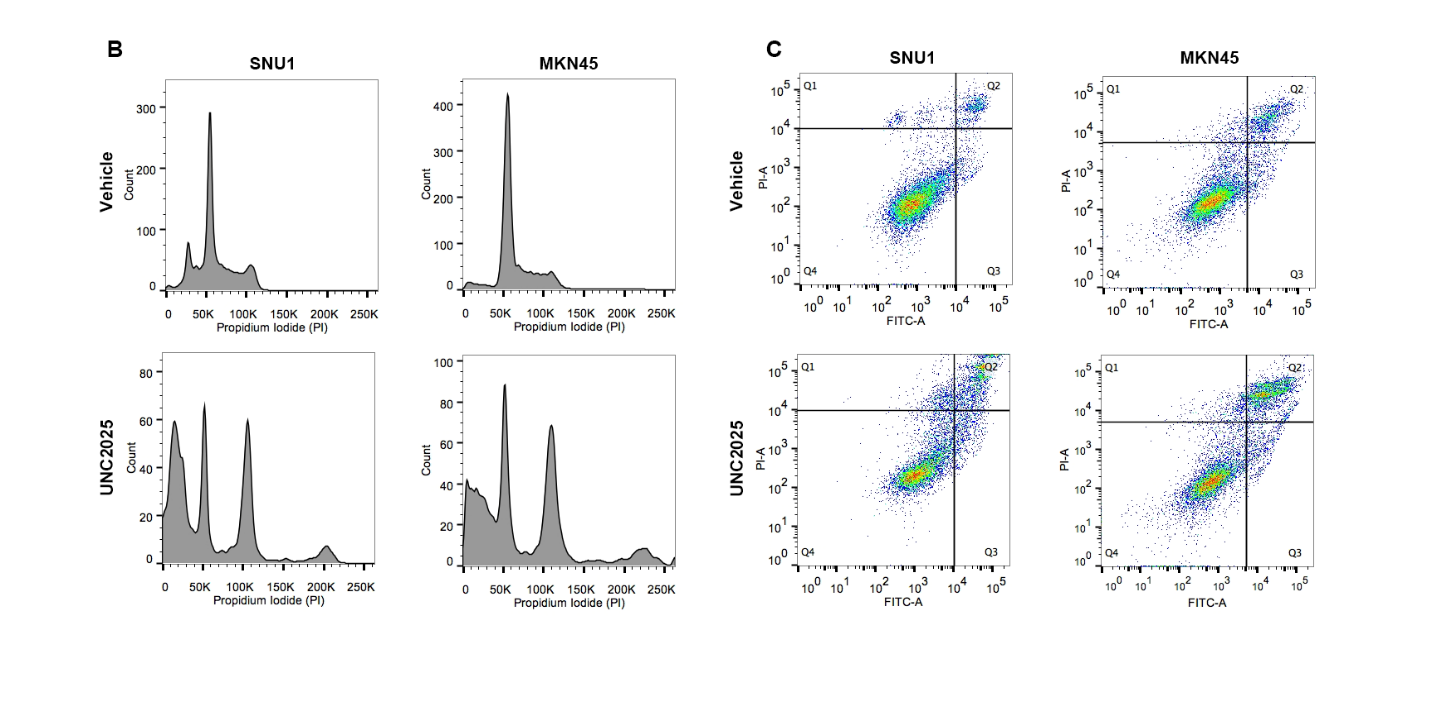


**Supplementary Figure S3: UNC2025 exhibits therapeutic value when used alone**

(**A**) IC_50_ level of UNC2025 was calculated using WST-1 assay in SNU1, SNU5 and MKN45 cell lines. (**B**) SNU1 and MKN45 cells were treated with IC_50_ concentration of UNC2025 and fixed in 70% ethanol overnight together with 50 µg/ml PI and 25 µg/ml RNase. The percentage of cells in subG1, G0/G1, S and G2/M phases as well as cells with polyploid were calculated using flow cytometry. Representative images were shown. (**C**) SNU1 and MKN45 cells were stained with Annexin V/PI after treated with IC_50_ concentration of UNC2025 and flow cytometric measurement was performed. Representative images were shown.

**Supplementary Figure 4**


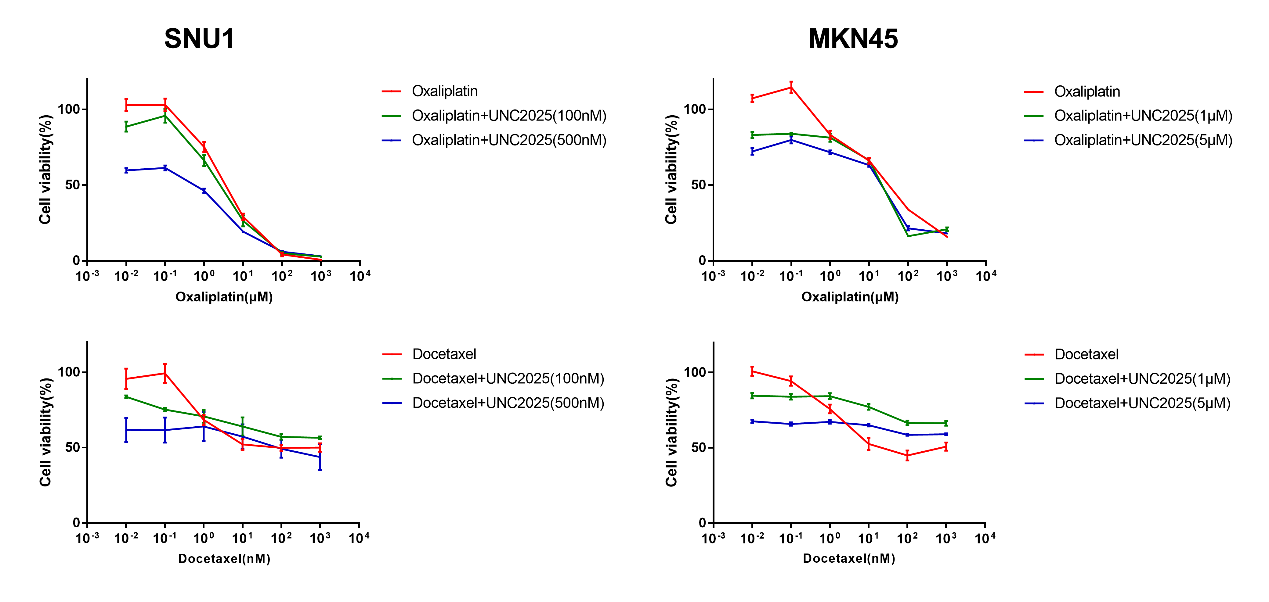


**Supplementary Figure S4:** **Treatment with oxaliplatin and docetaxel in combination with UNC2025**

Cells were seeded in 96 well plates at the density of 1*10^4^ and treated with different concentration of oxaliplatin or docetaxel in combination with UNC2025. Cell viability was detected using WST-1 assay. Relatively small concentration of UNC2025 (100nM for SNU1, 1μM for MKN45) did not change the response towards oxaliplatin and docetaxel.

**Supplementary Figure 5**


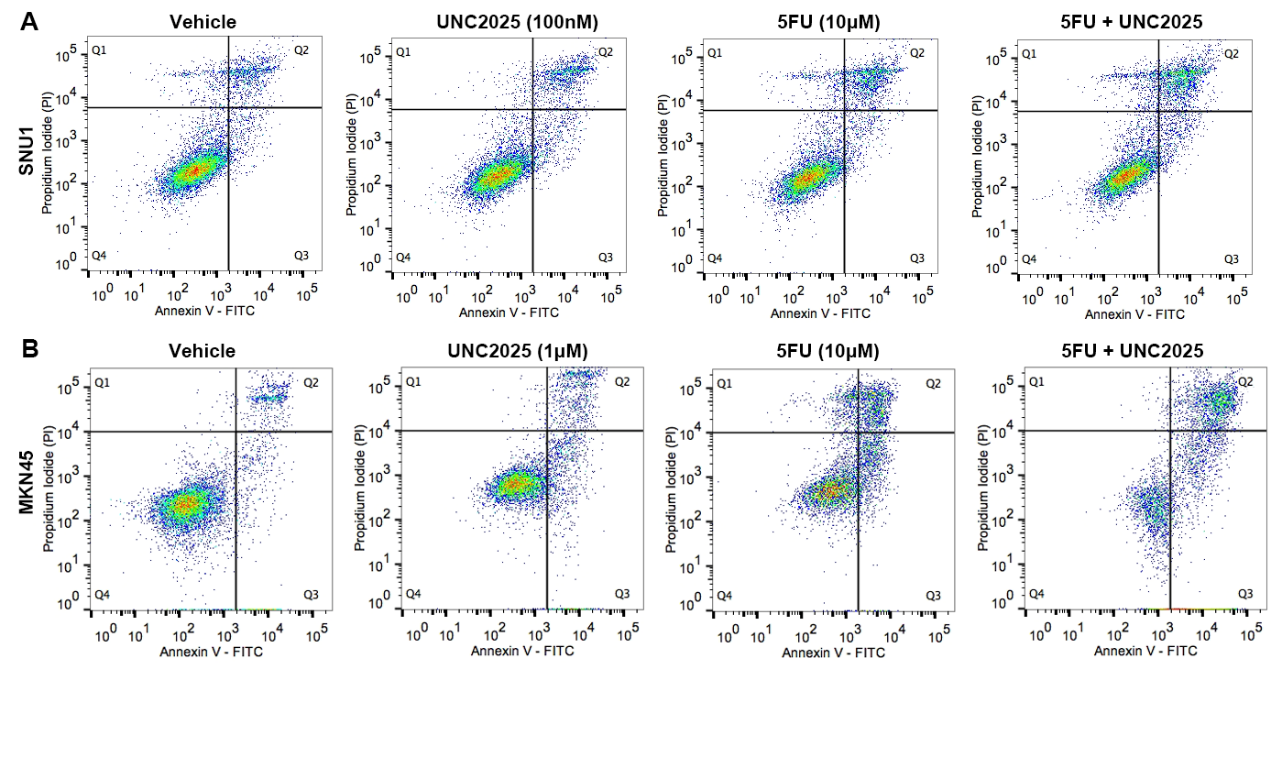


**Supplementary Figure S5:** **Increased level of apoptotic cells when combined 5FU with UNC2025**

SNU1 and MKN45 cells were stained with Annexin V/PI after treated with 5FU alone and in combination of UNC2025. Flow cytometry was performed. Representative images of SNU1 (**A**) and MKN45 (**B**) cells were shown.
