# Supplementary material for: Targeting the receptor tyrosine kinase MerTK shows therapeutic value in gastric adenocarcinoma
Source: Cancer Med. 2024 Mar 28;13(7):e6866. doi: 10.1002/cam4.6866 (PMC10974716; doi:10.1002/cam4.6866)
Supplement: Supplementary file 2 — Table S1. [file CAM4-13-e6866-s001.docx]

**Supplementary Table 1. Clinicopathological variables and MerTK expression in gastric cancer patients (n=140)**

| **Variables** | **Total** | **MerTK Expression** | |
| --- | --- | --- | --- |
|  |  | Low (H-Score＜98) | High (H-Score≥98) |
| **Age (years)** |  | | |
| ≤ 60 | 49(35.0%) | 41(37.6%) | 8(25.8%) |
| ＞60 | 91(65.0%) | 68(62.4%) | 23(74.2%) |
| **Gender** |  | | |
| Male | 100(71.4%) | 77(70.6%) | 23(74.2%) |
| Female | 40(28.6%) | 32(29.36%) | 8(25.8%) |
| **Clinical T Stage** |  | | |
| cT1 | 2(1.4%) | 2(1.8%) | 0(0.0%) |
| cT2 | 24(17.1%) | 18(16.5%) | 6(19.4%) |
| cT3 | 83(59.3%) | 64(58.7%) | 19(61.3%) |
| cT4 | 27(19.3%) | 22(20.2%) | 5(16.1%) |
| cTx | 4(2.9%) | 3(2.8%) | 1(3.2%) |
| **Clinical N Stage** |  | | |
| cN0 | 32(22.9%) | 24(22.0%) | 8(25.8%) |
| cN+ | 105(75.0%) | 83(76.2%) | 22(71.0%) |
| cNx | 3(2.1%) | 2(1.8%) | 1(3.2%) |
| **Clinical M Stage** |  | | |
| cM0 | 115(82.1%) | 94(86.2%) | 21(67.7%) |
| cM1 | 24(17.1%) | 15(13.8%) | 9(29.1%) |
| cMx | 1(0.7%) | 0 (0%) | 1 (3.2%) |
| **Grading** |  | | |
| G1 | 1(0.7%) | 1(0.9%) | 0(0.0%) |
| G2 | 32(22.9%) | 23(21.1%) | 9(29.0%) |
| G3 | 72(51.4%) | 55(50.5%) | 17(54.8%) |
| Gx | 35(25%) | 30(27.5%) | 5(16.1%) |
| **Neoadj. chemotherapy** |  | | |
| No | 44(31.4%) | 33(30.3%) | 11(35.5%) |
| Yes | 96(68.6%) | 76(69.7%) | 20(64.5%) |
| **Pathological T Stage** |  | | |
| pT1 | 15(10.7%) | 13(11.9%) | 2(6.4%) |
| pT2 | 21(15.0%) | 17(15.6%) | 4(12.9%) |
| pT3 | 72(51.4%) | 53(48.6%) | 19(61.3%) |
| pT4 | 29(20.7%) | 23(21.1%) | 6(19.4%) |
| pTx | 3(2.1%) | 3(2.8%) | 0(0%) |
| **Pathological N Stage** |  | | |
| pN0 | 48(34.3%) | 41(37.6%) | 7(22.6%) |
| pN1 | 22(15.7%) | 20(18.3%) | 2(6.5%) |
| pN2 | 24(17.1%) | 15(13.8%) | 9(29.0%) |
| pN3 | 45(32.1%) | 32(29.4%) | 13(41.9%) |
| pNx | 1(0.7%) | 1(0.9%) | 0(0%) |
| **Pathological M Stage** |  | | |
| pM0 | 114(81.4%) | 90(82.6%) | 24(77.4%) |
| pM1 | 26(18.6%) | 19(17.4%) | 7(22.6%) |
| **Tumor regression**  no vital tumor cells | 46(32.9%) | 35(32.1%) | 11(35.5%) |
| <10% vital tumor cells | 27(19.3%) | 26(23.8%) | 1(3.2%) |
| 10-50% vital tumor cells | 30(21.4%) | 27(24.8%) | 3(9.7%) |
| >50 vital tumor cells | 37(26.4%) | 21(19.3%) | 16(51.6%) |

T: primary site; N: regional lymph node; M: metastasis
