# Supplementary material for: Targeting the receptor tyrosine kinase MerTK shows therapeutic value in gastric adenocarcinoma
Source: Cancer Med. 2024 Mar 28;13(7):e6866. doi: 10.1002/cam4.6866 (PMC10974716; doi:10.1002/cam4.6866)
Supplement: Supplementary file 3 — Tables S2–S4. [file CAM4-13-e6866-s002.docx]

**Supplementary Table 2. Antibodies for immunohistochemistry staining**

| **Products** | **Company** |
| --- | --- |
| **Primary antibodies** |  |
| Anti-Ki67, Rabbit, Monoclonal | Abcam, ab15580 |
| Anti-MerTK, Rabbit, Monoclonal | Abcam, ab52968 |
| Anti-cleaved caspase-3, Rabbit, Polyclonal | Cell Signaling technology, 4970 |
| **Secondary antibodies** |  |
| Goat Anti-Rabbit IgG Antibody (H+L), Biotinylated | Vector, BA-1000 |

**Supplementary Table 3. Antibodies for western blot**

| **Products** | **Company** |
| --- | --- |
| **Primary antibodies** |  |
| Anti-Akt, Rabbit, Polyclonal | Cell Signaling technology, 9272 |
| Anti-Axl, Rabbit, Monoclonal | Abcam, ab227871 |
| Anti-Bax, Rabbit, Polyclonal | Cell Signaling technology, 2772 |
| Anti-Bcl-2, Rabbit, Monoclonal | Cell Signaling technology, 3498 |
| Anti-MerTK (phospho Y749+Y753+Y754), Rabbit, Monoclonal | Abcam, ab14921 |
| Anti-MerTK, Rabbit, Monoclonal | Abcam, ab52968 |
| Anti-p44/42 MAPK(Erk1/2), Rabbit, Monoclonal | Cell Signaling technology, 9102 |
| Anti-phospho-Akt (Ser473), Rabbit, Monoclonal | Cell Signaling technology, 4060 |
| Anti-phospho-p44/42 MAPK(Erk1/2) (Thr202/Ty204), Mouse, Monoclonal | Cell Signaling technology, 9106 |
| Anti-Tyro3, Rabbit, Monoclonal | Abcam, ab109231 |
| Anti-β-actin, Rabbit, Monoclonal | Cell Signaling technology, 4970 |
| **Secondary antibodies** |  |
| Goat pAb to Mouse IgG (HRP) | Abcam, ab97023 |
| Goat pAb to Rabbit IgG (HRP) | Abcam, ab6721 |

**Supplementary Table 4. sgRNAs for CRISPR/Cas9**

| **Names** | **Sequences (5'-3')** |
| --- | --- |
| human_sgMERTK_1_forward | CACCGCCAGGGCCCTTATCGCCCAT |
| human_sgMERTK_1_reverse | AAACATGGGCGATAAGGGCCCTGGC |
| human_sgMERTK_2_forward | CACCGTTACAGCTATCACTGAGGCA |
| human_sgMERTK_2_reverse | AAACTGCCTCAGTGATAGCTGTAAC |
| human_sgMERTK_3_forward | CACCGATCCCCGAAACTAAGCTGTC |
| human_sgMERTK_3_reverse | AAACGACAGCTTAGTTTCGGGGATC |
